# The Paradox Role of Regulatory T Cells in Ischemic Stroke

**DOI:** 10.1155/2013/174373

**Published:** 2013-10-31

**Authors:** Xiaomeng Xu, Min Li, Yongjun Jiang

**Affiliations:** Department of Neurology, Jinling Hospital, Nanjing University School of Medicine, Nanjing, Jiangsu 21002, China

## Abstract

The underlying mechanism of ischemic stroke is not completely known. Regulatory T cells (Tregs), a subset of T cells, play a pivotal role in the pathophysiological process of ischemic stroke. However, there is also controversy over the role of Tregs in stroke. Hence, the function of Tregs in ischemic stroke has triggered a heated discussion recently. In this paper, we reviewed the current lines of evidence to describe the full view of Tregs in stroke. We would like to introduce the basic concepts of Tregs and then discuss their paradox function in ischemic stroke. On one side, Tregs could protect brain against ischemic injury via modulating the inflammation process. On the other side, they exaggerated the insult by causing microvascular dysfunction. They also interfered with the neurological function recovery. In addition, the reasons for this paradox role would be discussed in the review and the prospective of the clinical application of Tregs was also included. In conclusion, Tregs contributed to the outcome of ischemic stroke, while more lines of evidence are needed to understand how Tregs regulate the immune system and influence the outcome of stroke.

## 1. Introduction

Stroke, of all cases ischemic stroke that accounts for more than 87% [1], is the leading cause of morbidity and permanent disability in adults [2], which results in severe social-economic burden worldwide [3] especially in developing countries such as China [4]. During the past decades, notable and multidisciplinary progress was made in the stroke mechanisms in order to reduce the burden of stroke. Among them, immune system plays a pivotal role in the pathophysiological process of ischemic stroke.

Traditionally, immune system and central nervous system have been thought of as two distinct entities [5], considering the anatomical and physiological obstacles including the existence of the blood-brain barrier [6], the lack of cerebral lymphatic vessels, and the inefficiency of microglia and astrocytes for antigen presentation to T cells [7]. However, recent data indicates that there is an active interaction between these two systems [8]. Researches in cerebrovascular field have focused on stroke-associated inflammatory processes [9], featured by the necrosis of cerebral tissue, breakdown of blood-brain barrier, excessive release of inflammatory intermediates, and infiltration of leukocyte. On one side, inflammation has been regarded as a hallmark of acute stroke [10] but on the other side it is proven to increase secondary infarct growth and delay neural function recovery [11]. Therefore, proper regulation of the stroke-associated inflammation is of vital importance in the neuroprotection and poses a potential therapeutic approach in post stroke management [12].

During post-stroke inflammation, T cells are recruited into the ischemic brain within 24 hours after stroke onset [13, 14] and are well accepted as a deleterious component that exaggerates brain injury [14]. However, the contribution of the different T cell subsets remains subtle [15]. Of note, regulatory T cells (Tregs) are renowned to play an indispensable part in immunoregulation and selftolerance with the capability to counteract overactivated immune response. In particular, a controversial dispute arose on the function of Tregs in the ischemic brain [15].

Based on a completed search carried out through databases Medline (source PubMed) and Web of Science without restriction of publication time or language, with the terms “regulatory T cells,” “T regulatory cells,” “Tregs,” and “stroke,” as well as further searches done by reviewing relevant references of review articles manually, this review was intended to present a comprehensive summary of current knowledge of Tregs involved in post-stroke inflammation and was mainly focused on preclinical studies exploring functional roles of Tregs.

## 2. A Brief Overview of Tregs

Tregs, a subset of T cells, play a crucial role in the suppression of excessive immune response, the maintenance of immunological selftolerance, and the preservation of immune homeostasis [16]. The deficiency of Treg function (e.g., owing to forkhead box P3, Foxp3, gene mutation) would evoke various autoimmune diseases, immunopathology, and allergy [17]. Tregs consist of many subpopulations, including natural Tregs, Th3, Tr1, CD8 Tregs, and natural killer Tregs (NK Tregs), which share a common characteristic of immunosuppressive capability but differ in surface markers and sites of formation. Among these subpopulations, natural Tregs that express CD4, CD25, and Foxp3 are most studied and well understood [18]. Natural Tregs are developmentally determined in the thymus as a distinct T cell subpopulation specialized for suppressive function; conversely, other subpopulations, known as inducible Tregs, are adaptively regulatory and acquire their regulatory functions following specific antigenic stimulation in particular cytokine milieus [19].

This suppressive T cell subset was recognized in the 1970s when Gershon and Kondo [20] discovered that T cells could not only promote but also dampen immune response, though these cells were referred to as “suppressor T cells” at that time. But further exploration was severely hampered by the failure to distinguish these suppressor T cells from other T cells. It was until 1995 that a specific surface marker, CD25 [21], was identified, enabling the isolation and identification of suppressor T cells. Henceforth, the subset gradually gained increasing attention and was renamed as “regulatory T cells” [22], and in 2001, CD4+CD25+ Tregs were identified in human by several independent studies [23–27]. Discovery of the functional role of transcription factor Foxp3, to date the most specific marker for Tregs, was another milestone in this field [22], which allowed the introduction of genetically engineered mouse model and therefore accelerated Tregs researches.

Despite the long history of Treg research, molecular basis for the suppression has not been definitively characterized so far and is the subject of intense research currently. In general, Tregs exert their suppressive ability via cell contact dependent and independent mechanisms. The cell contact dependent mechanism was substantiated by transwell experiments in which Tregs failed to suppress T cell activation across semipermeable membrane [28]. On one hand, Tregs were able to induce T cell cytolysis in a granzyme-mediated [29, 30] or a perforin-mediated [31, 32] manner. On the other hand, Tregs could inhibit effector T cell function via delivery of negative signals. For example, studies suggested that by concordant expression of CD39 and CD73, Tregs would generate adenosine that acts on the adenosine receptor on effector T cells to induce suppressive effects [33–35]; besides, Tregs were proved to directly transfer cyclic adenosine monophosphate (AMP), an inhibitory second messenger, through membrane gap junctions into effector T cells, leading to inhibited proliferation and interleukin-2 (IL-2) production [36]. In addition to the interaction with T cells, Tregs prompted dendritic cells (DCs) to express immunosuppressive molecules [37] and mitigate effector T cell activation by DCs [38, 39]. In contrast to the undisputable indispensability of cell contact in Treg function, cell contact independent mechanisms are more controversial. Interleukin-10 (IL-10) and transforming growth factor-*β* (TGF-*β*) were hypothesized to be involved in Treg mediated immunosuppression since Tregs could secrete these cytokines and these cytokines are irrefutably immunosuppressive, yet their contribution to the function of thymus-derived, naturally occurring Tregs is still a matter of debate [40]. In order to achieve the maximal regulatory activity of Tregs, IL-10 and TGF-*β* were critically required in vivo, yet meanwhile studies showed that neutralization of either IL-10 or TGF-*β* did not abrogate in vitro suppression [41, 42]; therefore, this hypothesis still requires more evidence to refine.

Based on the knowledge that Tregs play a vital role in the regulation and modulation of immune homeostasis, it is logical to presume that Treg dysfunction leads to disorders of immune system such as autoimmune diseases. Interestingly, recent studies demonstrated that Tregs were associated with vascular disease, including myocardial infarction [43], atherosclerosis [44], hypertension [45], and also stroke [46].

## 3. Regulatory T Cells in Acute Ischemic Stroke

### 3.1. Redistribution and Accumulation

Clinical observation suggested that the number of circulating Tregs would fall pronouncedly in the second day after stroke onset [47], followed by an increase on day 7 that lasted at least throughout week 3 [48]. This fluctuation gave rise to the hypothesis that perhaps during an acute phase after stroke, Tregs left the circulation and migrated to target tissues [12]. In order to clarify the post-stroke distribution of Tregs, animal studies were conducted thereafter.

In accordance with the previous findings in human beings, experimental results in murine models indicated an early reduction of Tregs in the periphery including blood and spleen. Stroke was known to cause a transient splenic atrophy, characterized by a dramatic declination of splenic size as well as the number of splenocytes within 48 hours after stroke, both of which would return to normal level by 96 hours [49]. Surprisingly, the splenic atrophy was accompanied with escalating Tregs with the cell count remaining stable at the 22-hour time point followed by an evident surge at the 96-hour time point [46]. Consistently, another study [50] substantiated the discovery and demonstrated that in an early stage, circulation of Tregs decreased significantly 24 hours after the cerebral ischemic insult and lasted 3 days before returning to normal level. Furthermore, the study found that in contrast to the variation in the peripheral number of Tregs within the ischemic sphere doubled upon the first day after stroke and kept increasing out to day 3, yet no Tregs were detected in the cerebral parenchyma until the fifth day [51]. All of the recruited Tregs were grouping in the infarct or peri-infarct areas and lingering within cerebral vascular lumen within 4 days. In a longer term, the escalating lasted out to 4 weeks and a trespass across brain vessel was observed on day 5 [52].

Collectively, all the findings illustrated an early redistribution and accumulation of Tregs within the ischemic spheres 4 days after stroke without intruding the blood-brain barrier until the fifth day, and the response lasted out to 4 weeks.

### 3.2. Function and Mechanism

Despite the ascending enthusiasm in Tregs, the functional roles of Treg after acute ischemic stroke remain elusive, with some studies suggesting a protective impact while others suggesting a negative result. Recently, intensified discussion over this controversy has been initiated again by the leading journal Stroke [53]. Here, we reviewed the recent publications to describe the full view of Tregs in postischemic stroke [54].

#### 3.2.1. Pros

Initially, it was found that natural Tregs were powerful inhibitors of atherosclerosis, which is an immunoinflammatory disease elicited by accumulation of lipids in the artery wall and the leading cause of stroke. Liesz et al. [55] demonstrated that Tregs might play a beneficial role in post-stroke injury by preventing secondary infarct growth against ischemic insult. They verified their findings via two distinct approaches: antibody-mediated Treg depletion and adoptive cell transfer. In the Treg depletion part, anti-CD25 mAb or PBS was administrated two days before performance of permanent MCAO and resulted in significantly enlarged infarcts in the Treg-depleted mice 7 days after the coagulation, together with markedly worsened neurological function. In the cell transfer part, total CD4+ T cells, Treg cells, or CD4+CD25− T cells were transferred into lymphocyte deficient Rag2^−/−^ mice, and those receiving CD4+CD25− cells alone showed markedly larger infarcts, indicating that deprivation of CD25+ Tregs might be associated with a worse outcome, in consistency with the Treg depletion experiment. In consistency with Liesz et al., Li et al. [51] explored the therapeutic effect of Treg adoptive strategy and revealed that post-stroke delivery of Tregs within 24 hours could markedly reduce ischemic stroke volume, accompanied with improved neurological functions, and the beneficial effects prolonged out to 4 weeks.

Even though the interaction between the brain and the immune system subsequent to ischemic stroke has not been documented until recently [7, 11], the functional role of Tregs in other pathological courses, including atherosclerosis [43], myocardial infarction [56], and ischemia/reperfusion injury in the liver [57] and kidney [58], has already been well addressed, and most of the literatures have ascribed the protective effect to a relief of excessive inflammatory response. Therefore, it is reasonable to attribute the neuroprotective ability of Tregs to a similar mechanism.

Firstly, Tregs can exert the anti-inflammatory effect by secreting the cytokine interleukin-10, which is widely acknowledged as anti-inflammatory cytokine (IL-10) [7]. The neuroprotective role of IL-10 was that noted decades ago for IL-10 could reduce brain injury following MCAO, whether administered intravenously or into lateral ventricle [29] and IL-10 knockout would result in enlarged infarcts [31]. In addition, Liesz et al. [55] confirmed the hypothesis in their Treg-depleted MCAO mouse model. On the basis of anti-CD25 mAb mediated Treg-depletion, exogenous IL-10 supplement would lead to a marked diminishment in infarct volume as compared with the controls. Interestingly, it was also noticed that only intracerebroventricular injection, but not intraperitoneal injection, would result in the positive outcome, in consistency with the local accumulation of Tregs during the acute phase after stroke and suggested that the anti-inflammatory effect of IL-10 seems to be achieved by a direct action on brain cells [12]. 

Besides, Tregs conferred protection against MCAO by blunting a rise in metalloproteinases-9 (MMP-9) [51]. MMPs have been thought to be involved in stroke pathogenesis and clinical reports indicated elevated serum level of MMP-9 among patients with ischemic stroke [38]. Stroke-induced MMP-9 production owing to neutrophil infiltration played an important role during the breakdown of blood-brain barrier [39] and hence promoted leukocyte infiltration and brain damage [37], whereas Treg adoptive treatment inhibited MMP-9 production in the blood and the brain as early as 1 day after ischemia [51]. Tregs might exert this inhibitory capability via cell-to-cell contact with neutrophils as Tregs cultured into the transwells lost their inhibition on MMP9 production. 

#### 3.2.2. Cons

In spite of all the positive contributions mentioned above, recent findings indicated a deleterious role of Tregs as well. As was shown in the experiment conducted by Kleinschnitz et al. [50], Tregs might exacerbate ischemic-reperfusion damage in a very early phase after stroke, that is, within 24 hours. This study accomplished Treg depletion via genetic modified mouse models, the DEREG mice, and in opposition to former discoveries, the authors found that, followed by 60 min MCAO, Treg depletion should result in a decrease in infarct volume with an improvement in neurological function, and the beneficial effect would last out to 4 days; accordingly, MRI of depletion group showed smaller infarcts on day 1 without any evident progression observed throughout 1 week, which excluded the probability for a secondary growth. Subsequently, the authors examined the influence of delayed Treg depletion. As a consequence, elimination of Tregs 1 day after 30 min MCAO was not relevant to secondary growth out to 1 week. Taken together, neither early nor delayed depletion was associated with a secondary growth in this study. Finally, in order to substantiate the discovery of the deleterious effects of Tregs, the authors conducted an adoptive experiment where Tregs were transferred to Rag2^−/−^ mice 1 day prior to 30 min MCAO, and evaluation of infarct volume on day 1 verified that Treg adoption contributed to revere infarct reduction in Rag^−/−^ mice.

Currently, the underlying mechanism for these unexpected impacts of Tregs is scarce; however, it was suggested that Tregs might promote neuronal damage by inducing microvascular dysfunction [15], since reduced amount of fibrin and higher level of cerebral blood flow were detected among the Treg-depleted mice within 24 hours after tMCAO, indicating better cerebral reperfusion and less tissue damage. During this phase after stroke, Tregs were recruited from the periphery to migrate into the ischemic spheres [52], especially within the infarct and peri-infarct area; however, the accumulating Tregs were unable to trespass blood-brain barrier but were lingering predominantly within cerebral vessels, suggesting a low probability to interact directly with parenchymal tissue but a higher possibility to disrupt the microvascular structure. In addition, inhibition of the communication between these lymphocytes and endothelium in Rag2^−/−^ mice transferred with CD4+CD25+ Tregs by anti-LFA-1 mAb was associated with reduced post-tMCAO damage, suggesting the involvement of intercellular adhesion molecule 1 (expressed on endothelium)/lymphocyte function-associated antigen-1 (expressed on T cells) pathway [50]. In conclusion, mechanisms acting at the brain-vasculature interface could be of pathologic relevance in the exacerbation caused by Tregs.

Furthermore, the immunosuppression caused by Tregs might compromise the benefits achieved by post-stroke inflammation such as tissue regeneration and repair [59]. Besides, whether the modulation of Treg cells after stroke lowered the threshold for infection poses another concern [12] although this hypothesis might be applicable to a long term outcome and irrelevant to the pathophysiological response during the very early stage reported by Kleinschnitz et al. [50].

#### 3.2.3. Neutral Findings

In addition to the studies above demonstrating either advantageous or deleterious impacts, current understanding of Tregs is presented with a number of researches of neutral findings as well, reporting that Tregs may not be relevant to the post-stroke outcome at all. 

*Ren et al. (2011)*. This study yielded to a neutral conclusion that Treg depletion did not affect stroke infarct volume. Instead of CD25-specific antibody, Treg depletion in this experiment was achieved via a genetic mouse model (DEREG mouse), allowing for the ablation of Foxp3+ Tregs with higher specificity. Sixty-minute temporary MCAO was conducted in the mice with or without Treg depletion to achieve lesions of around 50 mm^3^ in size. Infarcts volume and behavior of each group were assessed on the fourth day after MCAO, yet neither of them showed any significant difference.

*Gu et al. (2012)*. This study explored the post-ischemic function of all subsets of T cells, aiming at clarifying the protective or detrimental roles of distinctive subsets after stroke. In this study, CD8+ cytotoxic T cells, CD4+ Th1 cells, CD4+ Th2 cells, and Tregs were evaluated both in vivo and in vitro, by means of genetic knockout of each subset and coculture of neurons with splenocytes from each knockout. As was illustrated in this experiment, elimination of Treg did not influence the outcome, yet deficiency of Th1 would attenuate while Th2 knockout would aggravate inflammatory response in vivo and neuronal death in vitro.

*Stubbe et al. (2013)*. This study was mainly focused on the accumulation, proliferation, and function of endogenous Tregs in a late phase after stroke. By means of fluorescence activated cell sorting analysis and immunohistochemistry, the authors demonstrated that after MCAO, Tregs began to accumulate in the infarct and peri-infarct areas on day 7, and the ipsilesional accumulation persisted till day 30. Treg proliferation in the infarct areas increased throughout days 7 and 14 before its declination to a nearly normal level by day 30. Moreover, in order to clarify the late function of Tregs, 30 min MCAO was performed followed by anti-CD25 mAb administered on days 3 and 14. Infarct volume was measured by MRI on days 3 and 27, and neural function was evaluated by gait analysis on days 14 and 27, but no significant difference was observed between the delayed Treg depletion group and controls.

## 4. Discrepancies and Discussion

As mentioned above, Tregs yield to profound inconstancy. The controversial topic has triggered intensified debate and discussion recently. Outcome of current studies in murine model was summarized in Table 1. 

### 4.1. Whether Endogenous Tregs Depletion Benefits or Exacerbates the Outcome

One of the major discrepancies posed by Liesz et al. [55] and Kleinschnitz et al. [50] is whether endogenous Tregs are relevant to secondary infarct growth after stroke. Liesz et al. proved that Treg depletion was associated with secondary infarct growth by day 7, although these effects would not become evident within the first 3 days, which was consistent with the neutral report from Ren et al. [60] and Gu et al. [61], whereas in the experiment conducted by Kleinschnitz et al., Treg depletion led to a better outcome within 24 hours and no progression occurred in both groups out to 1 week.

A plausible explanation for the inconsistency may be attributed to the ischemic duration and measured time because specific cell types of the immune system might be differentially relevant in different models of stroke [52]. The most part of the study conducted by Liesz et al. was based on a permanent MCAO mouse model inducing cortical infarction around 15 mm^3^ in size, while in the experiment reported by Kleinschnitz et al., a 60 min MCAO was adopted, resulting in infarcts around 50 mm^3^ involving cortical and subcritical tissue. In support of this assumption, Liesz et al. repeated their experiment in 30 min and 90 min temporary MACO models and proved that the effects of CD25+ Treg depletion were evident in mice with small infarcts (~15 mm^3^, after 30 min MCAO), but not in mice with larger damage (>100 mm^3^ after 90 min occlusion). Moreover, experiments in animal models with lesions of intermediate volumes (~50 mm^3^ after 60 min MCAO) [60, 61] failed to illustrate a significant result.

Secondly, the disagreement posed by Liesz et al. and Kleinschnitz et al. may originate from different method of Treg depletion. Liesz et al. achieved the depletion via anti-CD25 mAb while Kleinschnitz et al. introduced a genetic mouse model. Comparatively, the latter model conferred to a higher specificity as CD25 was also upregulated on activated T cells [40], and anti-CD25 mAb would therefore block CD25+ T cells other than Tregs to confound the outcome. In addition, the existence of CD25-negative Tregs subpopulations limits the interpretation of the data [15].

### 4.2. Whether Exogenous Treg Adoption Benefits or Exacerbates Outcome

Administration of exogenous Tregs induces another paradox. Li et al. proved that the adoption within 24 hours after the ischemic onset would exert a therapeutic effect that occurred by day 3 and lasted up to 28 days while Kleinschnitz et al. implemented the adoption 1 day before MCAO and ended up with evidently enlarged lesions in the transferred group by day 1.

This discrepancy might be justified by multiple reasons. Firstly, the quantities of Tregs delivered differed remarkably between these two protocols. Li et al. recommended a therapeutic dosage of 2 × 10^6^/mouse and noted that a concentration <1 × 10^6^ cells/mouse would fail to offer any early protection at all [53], whereas Kleinschnitz et al. used an even lower concentration of 7.5 × 10^5^/mouse. Moreover, the different timing of Treg delivery might be another probable interpretation of the divergence as well. Li et al. transferred the cells after the ischemic attack while Kleinschnitz et al. administered Tregs one day before stroke, and the preischemic increase of Tregs might be counterproductive, since the immune milieu before stroke may not be permissive for proper Treg action [53], and the delivery itself may disrupt the natural balance of immune system. Finally, these two studies focused on different stages after stroke. The former experiment emphasized the delayed effect in a long term with an observation period for a month while the latter concentrated on the short term efficacy within an acute phase of 24 hours and this divergence may count for the disagreement to a certain extent.

## 5. Prospective and Conclusion 

Current data is gradually revealing the contribution and mechanism of Tregs in post-stroke inflammation. Tregs were proved to respond to acute cerebral ischemic insult and undergo a rapid redistribution and accumulation into the ischemic sphere, implicating a potential involvement in the post-stroke inflammation. Additionally, depletion and adoption of Tregs had an influence on the neurological function, for better or for worse in rodent models.

However, current lines of evidence are faced with high heterogeneities in both methodology and results, and current opinions have not reached a consensus about whether Tregs are beneficial or detrimental in cerebral ischemic injury, implicating that investigation on this topic still lies in its infancy [53]. Infarct size in murine models, method of Treg depletion, timing, cell concentration, Tregs delivery, and observation period might intervene with the results. With the purpose to settle the contradiction, further attempt would be devoted to the clarification of the exact role of Tregs as well as the exploration of a rationale for the inconstant experimental outcome via identification of potential interfering factors and establishment of standard models.

Furthermore, Tregs have an innovative direction for clinical application in the treatment of ischemic stroke. Li et al. [51] suggested a promising therapeutic approach of post-stroke Treg delivery, although both its safety and efficacy required further elaboration. Additionally, the advantage of a protective effect was disputed by Kleinschnitz et al. [50], indicating that before the translation into clinical application, the controversy over an exact role of Tregs should be settled, as well as a defined timing and concentration of delivery. Future studies also will address whether the modulation of Treg cells after stroke would lead to poststroke immunodeficiency and make subjects vulnerable to secondary infection, a concern in previous clinical trials of immunomodulatory therapies in stroke [12]. Finally, the physiological differences between murine and human should be taken into careful consideration before making clinical interpretations. 

In conclusion, we need to know more to elaborate the functional role of Tregs, to understand how to regulate the immune system, and to improve outcome after stroke.

## Figures and Tables

**Table 1:** Outcome of current studies in murine model.

Study (year)	Outcome	Infarct volume	Treg depletion	Treg adoption	Major findings
Liesz et al. (2009) [55]	Protective	<30 mm^3^	Anti-CD25 mAb	None	Larger infarcts and worse neurological function in depletion group on day 7, but not day 1 or 3
			None	Total CD4+ T cells, Tregs or CD4+CD25− T cells to RAG2^−/−^ mice; 8∗10^6^/mouse; 7 days before MCAO	Larger infarcts in CD4+CD25− group on day 7

Ren et al. (2011) [60]	Neutral	~50 mm^3^	DEREG mouse (DT before MCAO)	None	No significant difference associated with depletion within 4 days after MCAO

Li et al. (2012) [51]	Protective	20–60 mm^3^	None	Tregs to wide type; 2∗10^6^/mouse; the 2nd, 6th, and 24th hours after reperfusion	Reduced infarcts and better neurological function in adoption group out to day 28
			Anti-CD25 mAb	Tregs to wide type; 2∗10^6^/mouse; the 2nd, 6th and 24th hours after reperfusion	

Stubbe et al. (2013) [52]	Neutral	~40 mm^3^	Anti-CD25 mAb	None	No significant difference associated with depletion

Kleinschnitz et al. (2013) [50]	Deleterious	60–90 mm^3^	DEREG mouse (DT before MCAO)		Smaller infarcts and better neurological function in depletion group on days 1 and 4; no secondary infarct growth associated with depletion by day 7
		5–10 mm^3^	DEREG mouse (DT after MCAO)		No secondary infarct growth associated with depletion by day 7
		5–10 mm^3^	DEREG mouse (DT before MCAO)	CD4+CD25+Tregs or CD4+CD25− T cells or CD4+CD25+FoxP3+ Tregs or CD4+CD25− FoxP3− T cells to RAG2^−/−^ mice; 7.5∗10^5^/mouse; 1 day before MCAO	Larger infarcts in all 4 adoption groups than Rag^−/−^ group; no difference between any adoption group and Rag^+/+^ group

Gu et al. (2012) [61]	Neutral			Ebi3 KO	No significant difference associated with depletion within 2 days after MCAO
